# Protective effects and possible mechanisms of matrine-type alkaloids (matrine and oxymatrine) in animal models of ulcerative colitis: a preclinical systematic review and meta-analysis

**DOI:** 10.3389/fphar.2026.1870457

**Published:** 2026-07-24

**Authors:** Shuqing Lu, Hanjian Yang, Shiying Wang, Jiahui Ye, Suiping Huang, Linkun Cai

**Affiliations:** 1 Department of Gastroenterology, The Second Affiliated Hospital of Guangzhou University of Chinese Medicine, Guangzhou, Guangdong, China; 2 Department of Colorectal Surgery, Clinical Oncology School of Fujian Medical University, Fujian Cancer Hospital, Fuzhou, Fujian, China; 3 College of International Education, Guangxi University of Chinese Medicine, Nanning, Guangxi, China; 4 Guangdong Provincial Key Laboratory of Clinical Research on Traditional Chinese Medicine Syndrome, Guangzhou, Guangdong, China

**Keywords:** inflammation, matrine, meta-analysis, oxymatrine, preclinical study, ulcerative colitis

## Abstract

**Background:**

Matrine and oxymatrine are bioactive alkaloids from Sophora species with reported anti-inflammatory and antioxidant effects in ulcerative colitis (UC). However, their overall efficacy has not been systematically evaluated.

**Methods:**

Databases including PubMed, EMBASE, Web of Science, Scopus, CNKI, Wanfang, VIP, and SinoMed were searched to January 2026. Animal studies using matrine or oxymatrine monotherapy in UC models were included. Effect sizes were calculated as standardized mean differences (SMDs) with 95% confidence intervals (CIs).

**Results:**

Thirteen studies were included. Compared with controls, matrine-type alkaloids significantly reduced histopathological scores (SMD = −3.47, 95% CI: −4.83 to −2.11) and decreased Disease Activity Index (DAI) at 3 days (SMD = −2.92, 95% CI: −4.27 to −1.57) and 7 days (SMD = −4.76, 95% CI: −6.30 to −3.23), but not at 14 days (SMD = −1.01, 95% CI: −2.22 to 0.20). Body weight increased at 7 days (SMD = 2.76, 95% CI: 1.95–3.57) and 14 days (SMD = 2.41, 95% CI: 0.12–4.69), and colon length was also increased (SMD = 2.09, 95% CI: 1.55–2.64). Matrine-type alkaloids reduced TNF-α (SMD = −2.88, 95% CI: −3.50 to −2.26), IL-6 (SMD = −2.91, 95% CI: −4.64 to −1.17), IL-1β (SMD = −2.73, 95% CI: −4.27 to −1.19), and MDA levels (SMD = −3.06, 95% CI: −4.47 to −1.65), while increasing SOD activity (SMD = 2.63, 95% CI: 1.37–3.89). Subgroup analyses suggested generally similar directions of effect across species and model types. The pooled effect estimate appeared larger in the oxymatrine subgroup than in the matrine subgroup, but this indirect comparison should be interpreted cautiously.

**Conclusion:**

Matrine-type alkaloids may exert protective effects in UC animal models by improving histological damage, reducing disease activity, and modulating inflammatory and oxidative stress pathways. These findings suggest their potential as multi-target therapeutic agents, although further high-quality studies are required for clinical translation.

**Systematic Review Registration:**

https://www.crd.york.ac.uk/PROSPERO/view/CRD420261376235, identifier CRD420261376235.

## 1 Introduction

Ulcerative colitis (UC) is a chronic, relapsing inflammatory bowel disease characterized by diffuse and continuous inflammation of the colonic mucosa, typically starting in the rectum and extending proximally (Raine et al., 2022; Bu et al., 2025). Although the exact pathogenesis of UC remains incompletely understood, it is generally considered to involve complex interactions among genetic susceptibility, impaired intestinal epithelial barrier function, dysregulated immune homeostasis, and alterations in the gut microbiota (Caba et al., 2025). In 2023, the global prevalence of UC was estimated to be approximately 5 million cases, with an increasing incidence worldwide (Le Berre et al., 2023). Persistent mucosal inflammation in UC not only leads to clinical manifestations such as diarrhea, hematochezia, and abdominal pain, but may also progress to severe colitis, colectomy, and an increased risk of colorectal cancer, thereby substantially impairing patient prognosis and quality of life (Dubinsky et al., 2023; Zhang and Gan, 2021).

Current therapeutic strategies for UC mainly include 5-aminosalicylic acid (5-ASA) preparations, corticosteroids, immunosuppressants, biologic agents, and small-molecule targeted therapies (Na et al., 2022; Cichewicz et al., 2023). Although these treatments have achieved considerable progress in inducing and maintaining remission, several limitations remain (Eder et al., 2023). On the one hand, a proportion of patients exhibit inadequate response or develop secondary loss of response, resulting in high relapse rates (Savelkoul et al., 2023; Matsuoka et al., 2025). On the other hand, long-term use of corticosteroids is associated with serious adverse effects, while biologics and small-molecule agents are limited by risks of infection, immunogenicity, and high treatment costs (Cichewicz et al., 2023; Khan et al., 2015). In addition, most current therapies target single or limited pathways, making it difficult to comprehensively address the complex, multi-pathway pathophysiology of UC (Bu et al., 2025; Na et al., 2022). Therefore, the development of novel therapeutic strategies with multi-target regulatory potential and favorable safety profiles is of great importance for improving the long-term outcomes of UC.

Matrine and oxymatrine are quinolizidine alkaloids primarily derived from plants of the genus Sophora (You et al., 2020; Fischer et al., 2024). Oxymatrine is the N-oxide derivative of matrine. As representative compounds of matrine-type alkaloids, they share highly similar chemical structures and may undergo metabolic interconversion *in vivo* (Yang et al., 2009). Previous studies have demonstrated that these compounds possess significant anti-inflammatory, immunomodulatory, and antioxidant properties, and can regulate inflammatory responses through multiple signaling pathways (Lin et al., 2022). In animal models of ulcerative colitis, matrine and oxymatrine have been reported to exert protective effects via various mechanisms, including inhibition of pro-inflammatory cytokine release, modulation of immune cell function, restoration of intestinal barrier integrity, and regulation of gut microbiota homeostasis (Li et al., 2019; Yao et al., 2021; Liu et al., 2022). However, existing evidence is largely derived from independent preclinical studies with considerable heterogeneity in model types, dosing regimens, and intervention durations, limiting a comprehensive evaluation of their overall efficacy and underlying mechanisms. Therefore, the present study aimed to systematically assess the protective effects of matrine-type alkaloids in animal models of UC through a preclinical systematic review and meta-analysis, and to further elucidate their potential mechanisms, thereby providing a theoretical basis for future translational research.

## 2 Materials and methods

This systematic review and meta-analysis was registered in the PROSPERO database (No: CRD420261376235) and was conducted and reported in accordance with the PRISMA 2020 statement.

### 2.1 Literature search

A systematic literature search was conducted in the following English-language databases: PubMed, EMBASE, Web of Science, and Scopus, as well as Chinese-language databases including China National Knowledge Infrastructure (CNKI), Wanfang Database, VIP Database, and SinoMed. The search covered all records from database inception to January 2026. The search terms included “ulcerative colitis,” “colitis,” “matrine,” “oxymatrine,” “Sophora flavescens,” “animal,” and “preclinical study.” In addition, the reference lists of all included studies were manually screened to identify any potentially relevant articles that might have been missed in the initial search. The complete search strategies for all databases are provided in Supplementary Table S1.

### 2.2 Eligibility criteria and study selection

The eligibility criteria were established *a priori* according to the registered review protocol and were structured using the PICOS framework, comprising population, intervention, comparator, outcomes, and study design. Specifically, the population was defined as *in vivo* animal models of ulcerative colitis; the intervention was matrine, oxymatrine, or matrine-type alkaloids administered as monotherapy; the comparator was vehicle treatment, no treatment, or model control; the outcomes included predefined indicators of colitis severity, histopathological injury, inflammatory response, and oxidative stress; and the study design was limited to controlled *in vivo* animal experiments.

The inclusion criteria were as follows (Raine et al., 2022): study type: controlled *in vivo* experimental studies using animal models of ulcerative colitis (UC), including dextran sulfate sodium (DSS)-, trinitrobenzene sulfonic acid (TNBS)-, or acetic acid-induced colitis models (Bu et al., 2025); intervention: administration of matrine, oxymatrine, or matrine-type alkaloids as monotherapy, with clearly reported dosage, route of administration, and duration of intervention (Caba et al., 2025); comparator group: receiving vehicle treatment, no treatment, or model control; and (Le Berre et al., 2023) outcomes: reporting at least one of the following predefined outcomes, including Disease Activity Index (DAI), histopathological score, body weight change, colon length, inflammatory cytokines (IL-1β, IL-6, TNF-α), and oxidative stress indicators (MDA, SOD).

The exclusion criteria were as follows (Raine et al., 2022): non-original studies or studies with insufficient extractable data (Bu et al., 2025); clinical studies, *in vitro* experiments, reviews, case reports, or study protocols (Caba et al., 2025); studies in which the intervention did not involve matrine, oxymatrine, or matrine-type alkaloids, or lacked clear information on dosage and treatment regimen; and (Le Berre et al., 2023) studies involving combination therapies in which the independent effects of matrine-type alkaloids could not be determined.

### 2.3 Data extraction

Two reviewers independently performed data extraction using standardized, pre-piloted data collection forms. Any discrepancies were resolved through discussion or consultation with a third reviewer. The following information was extracted from each eligible study: first author and year of publication; basic characteristics of the animals (species, sex, body weight, and sample size); modeling methods and model types; details of the intervention (compound type, dosage, route of administration, frequency, and duration); control group conditions; outcome measures; and potential mechanisms of action. For studies involving multiple eligible dosage groups sharing the same control group, a representative intervention group was selected according to prespecified criteria to avoid double-counting of the control group. When the original study clearly identified a recommended or primary effective dose, this dose group was selected. If no such information was provided, the dose group most consistent with the main experimental comparison reported by the original authors was used. This approach was predefined and applied consistently during data extraction. For studies in which data were presented only in graphical form, numerical values were extracted using image analysis software. When essential data were missing or unclear, the corresponding authors were contacted where possible. If the required data could not be obtained, the relevant outcome was excluded from the quantitative synthesis but retained for qualitative description when appropriate. When data were reported as SEM, they were converted to SD using standard formulas. All procedures were conducted in accordance with PRISMA guidelines.

### 2.4 Quality assessment

The methodological quality of the included studies was evaluated using the SYRCLE risk of bias tool for animal studies. Each domain was classified as “low risk,” “high risk,” or “unclear risk.” The assessment was conducted independently by two reviewers, and any disagreements were resolved through discussion.

### 2.5 Statistical analysis

Continuous variables were expressed as mean ± standard deviation (SD) or mean ± standard error of the mean (SEM) and organized using Microsoft Excel. Statistical analyses were performed using Review Manager (RevMan) version 5.3 and Stata version 16.0. Effect sizes were calculated as standardized mean differences (SMDs) with 95% confidence intervals (CIs), and a p value <0.05 was considered statistically significant. Heterogeneity among studies was assessed using the I^2^ statistic. A fixed-effect model was applied when I^2^ < 50%, whereas a random-effects model was used when I^2^ ≥ 50%, indicating substantial heterogeneity. To explore potential sources of heterogeneity, subgroup analyses were planned according to compound type, animal species, model type, sample size, dose category, route of administration, intervention duration, and risk-of-bias level when sufficient studies were available. Subgroup analyses were not performed when the number of studies within a given outcome or subgroup was too small to provide reliable and interpretable estimates. Sensitivity analyses were performed by sequentially omitting individual studies to evaluate the robustness of the pooled results. Additional sensitivity analyses were considered to assess whether the pooled estimates were influenced by studies with distinct methodological or intervention characteristics when sufficient data were available. Publication bias was planned to be assessed for outcomes including 10 or more studies using funnel plots and Egger’s regression test. Funnel plots were visually inspected for asymmetry, and a p value <0.05 in Egger’s test was considered indicative of potential publication bias.

## 3 Results

### 3.1 Study selection

**FIGURE 1:**
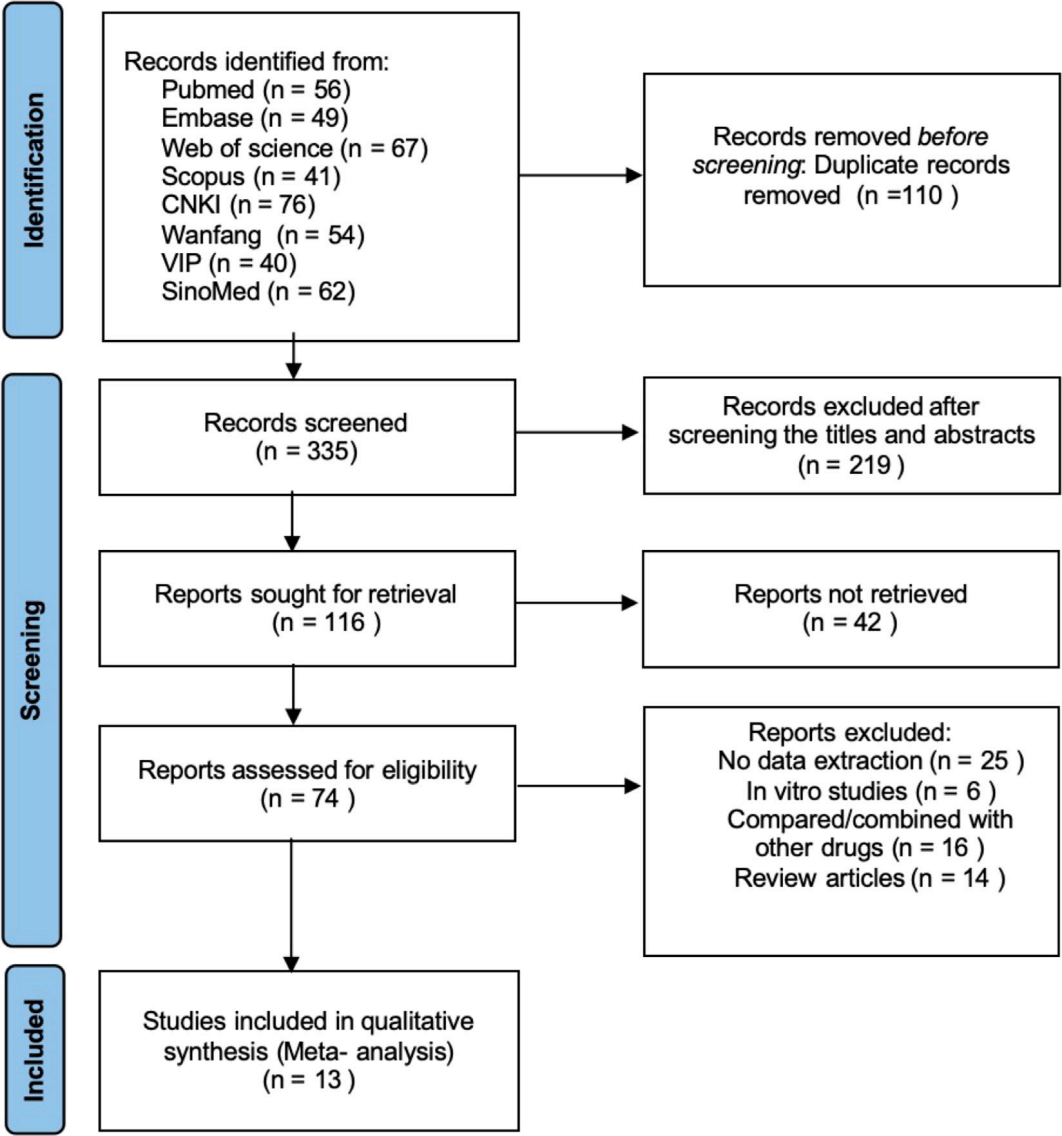
Flow diagram of study selection.

A total of 445 records were identified through the initial search. After duplicate removal and title/abstract screening, 116 reports were sought for full-text retrieval, of which 4 could not be retrieved. The remaining 112 reports were assessed for eligibility, and 99 were excluded. Ultimately, 13 studies were included in the meta-analysis (Figure 1).

### 3.2 Characteristics of the included studies

This meta-analysis included 13 (Yao et al., 2021; Zheng et al., 2001; Zhendong and Yang, 2011; Qing Tang et al., 2012; Yongai Xiong et al., 2012; Yifan Wang, 2019; Zheng, 2019; Yan et al., 2020; Feihao et al., 2023; Derong et al., 2023; Mao et al., 2024a; Li et al., 2025; Sun et al., 2025) animal studies published between 2001 and 2025 that evaluated the protective effects of matrine-type alkaloids in ulcerative colitis models. Among the included studies, DSS-induced colitis was the most commonly used model (n = 7), followed by TNBS-induced colitis (n = 4), with one study using an immune-sensitized TNBS/ethanol-induced colitis model and one study using a damp-heat combined with DSS-induced colitis model. The experimental animals mainly included Sprague–Dawley rats (n = 4) and C57BL/6 mice (n = 4), followed by BALB/c mice (n = 2), Wistar rats (n = 1), ICR mice (n = 1), and one study using mice with the strain not reported. With respect to sex, most studies used male animals (n = 10), while two studies used both males and females, and one study did not report sex. Regarding test compounds, oxymatrine was investigated in 7 studies and matrine in 6 studies. Oral gavage was the predominant route of administration (n = 10), followed by intramuscular injection (n = 2) and intraperitoneal injection (n = 1). The duration of treatment ranged from 8 to 65 days. Detailed characteristics of the included studies are summarized in Table 1.

**TABLE 1:** Characteristics of the included studies.

First author (year)	Species	Gender	Age	Body weight	Induction of UC	Sample size		Compound	Intervention		Methods of administration	Duration of study (d)
			​	Range		IG	CG		IG	CG		
Zheng (2001)	SD rats	male	Not reported	50–55 g	DSS-induced colitis model	8	8	matrine	63 mg/kg	NS	intramuscular injection	12
Zhong (2011)	SD rats	male	8–12 weeks	180–220 g	TNBS-induced colitis model	10	10	matrine	180 mg/kg	NS	oral gavage	21
Tang (2012)	SD rats	male	Not reported	250 ± 20 g	TNBS-induced colitis model	8	7	oxymatrine	63 mg/kg	NS	intramuscular injection	16
Xiong (2012)	SD rats	male and female (1:1)	Not reported	200 ± 20 g	TNBS-induced colitis model	10	10	oxymatrine	180 mg/kg	NS	oral gavage	22
Wang (2019)	BALB/c mice	male	Not reported	18–22 g	DSS-induced colitis model	8	8	oxymatrine	50 mg/kg	PBS	intraperitoneal injection	8
Zheng (2019)	mice	male and female (1:1)	Not reported	18–22 g	TNBS-induced colitis model	10	10	oxymatrine	50 mg/kg	Not reported	oral gavage	8
Yan (2020)	Wistar rats	male	Not reported	200 ± 10 g	immune-sensitized TNBS/ethanol-induced colitis model	15	25	matrine	120 mg/kg	drinking water	oral gavage	31
Yao (2021)	C57BL/6 mice	male	6–8 weeks	Not reported	DSS-induced colitis model	5	5	matrine	20 mg/kg	equal volume of water	oral gavage	8
Yu (2023)	BALB/c mice	male	Not reported	Not reported	DSS-induced colitis model	10	10	oxymatrine	100 mg/kg	Not reported	oral gavage	8
Xu (2023)	C57BL/6 mice	male	8–10 weeks	20 ± 2 g	DSS-induced colitis model	10	10	oxymatrine	200 mg/kg	Not reported	oral gavage	11
Mao (2024)	C57BL/6 mice	Not reported	6 weeks	Not reported	DSS-induced colitis model	6	6	matrine	20 mg/kg	NS	oral gavage	15
Li (2025)	C57BL/6 mice	male	8 weeks	Not reported	damp-heat combined with DSS-induced colitis model	12	12	oxymatrine	100 mg/kg	vehicle control	oral gavage	65
Sun (2025)	ICR mice	male	6–8 weeks	Not reported	DSS-induced colitis model	8	8	matrine	200 mg/kg	equal volume of water	oral gavage	15

### 3.3 Risk of bias of included studies

**FIGURE 2:**
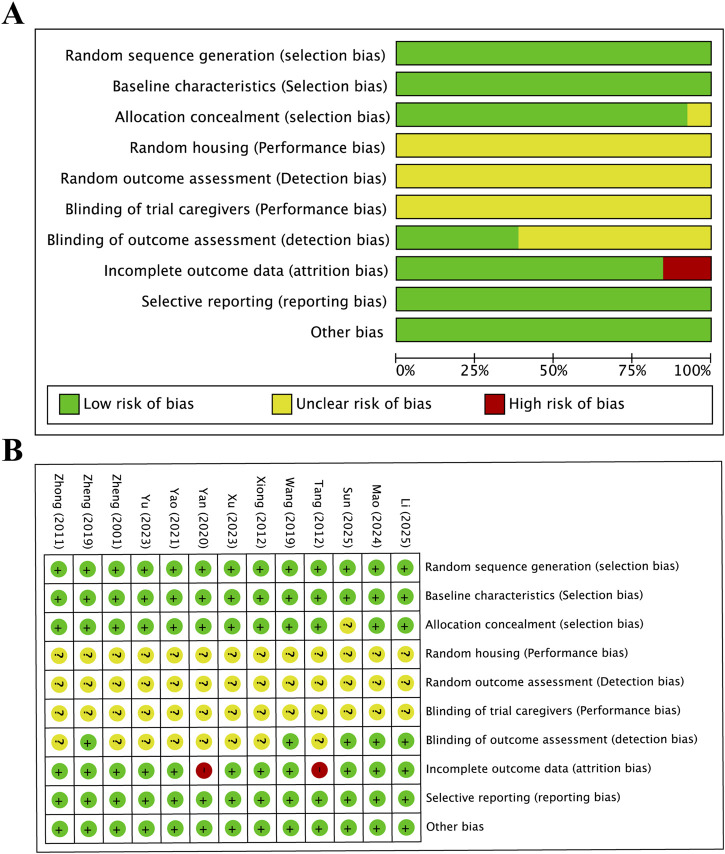
Risk of bias assessment of included studies. **(A)** Risk of bias graph showing the proportion of studies with low, unclear, and high risk of bias across different domains. **(B)** Risk of bias summary showing the risk of bias assessment for each included study.

The risk-of-bias assessment results are summarized in Figure 2. Overall, the included studies showed variable methodological quality. Although some domains, such as incomplete outcome data and selective outcome reporting, were generally judged as low risk, many studies were rated as unclear risk in key methodological domains because of insufficient reporting. In particular, details regarding random housing, blinding of caregivers and investigators, random outcome assessment, and blinding of outcome assessment were frequently not clearly described. These unclear-risk judgments indicate that incomplete reporting of randomization and blinding procedures may have introduced potential bias and reduced confidence in the reliability of the pooled evidence. Therefore, the findings of this meta-analysis should be interpreted with appropriate caution.

### 3.4 Meta-analysis

#### 3.4.1 Histopathological score

**FIGURE 3:**
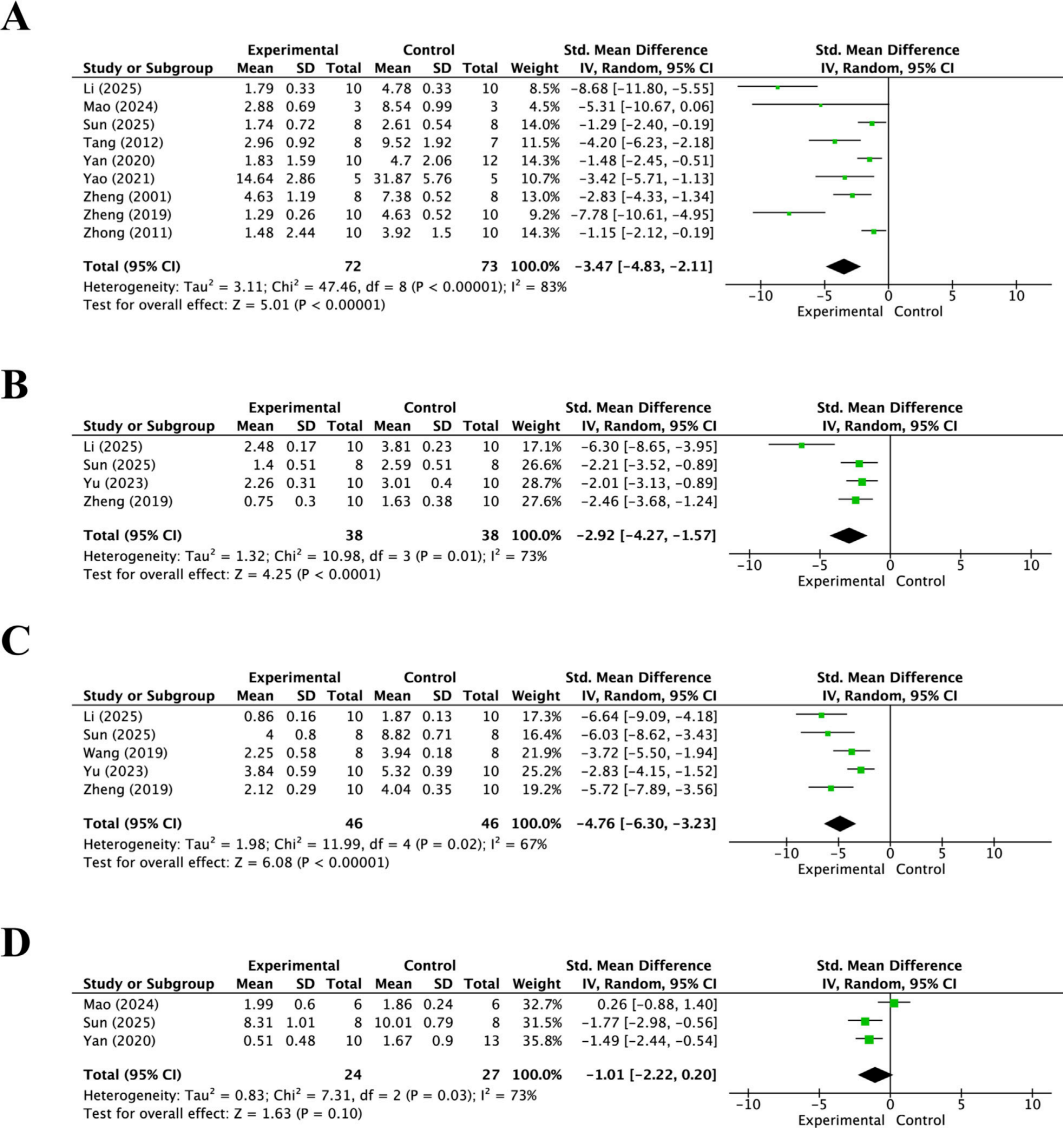
Effects of matrine-type alkaloids on histopathological score and Disease Activity Index (DAI) in animal models of ulcerative colitis. **(A)** Forest plot showing the effect of matrine-type alkaloids on histopathological scores. **(B)** Forest plot showing the effect of matrine-type alkaloids on DAI at 3 days. **(C)** Forest plot showing the effect of matrine-type alkaloids on DAI at 7 days. **(D)** Forest plot showing the effect of matrine-type alkaloids on DAI at 14 days.

Nine studies were included in the analysis of the impact of matrine-type alkaloids on histopathological scores in ulcerative colitis models. The results showed that, compared with the control group, the matrine-type alkaloid treatment group exhibited significantly reduced histopathological scores (SMD = −3.47, 95% CI = −4.83 to −2.11, p < 0.001) (Figure 3A).

#### 3.4.2 DAI

A total of 12 studies were included in the analysis of DAI. Among these, 4 studies measured outcomes at 3 days, 5 at 7 days, and 3 at 14 days. The results showed that, compared with the control group, the matrine-type alkaloid treatment group exhibited significantly reduced DAI scores at 3 days (SMD = −2.92, 95% CI = −4.27 to −1.57, p < 0.001) and 7 days (SMD = −4.76, 95% CI = −6.30 to −3.23, p < 0.001). However, no significant difference was observed at 14 days (SMD = −1.01, 95% CI = −2.22 to 0.20, p = 0.10) (Figures 3B–D).

#### 3.4.3 Effects on inflammatory cytokines

**FIGURE 4:**
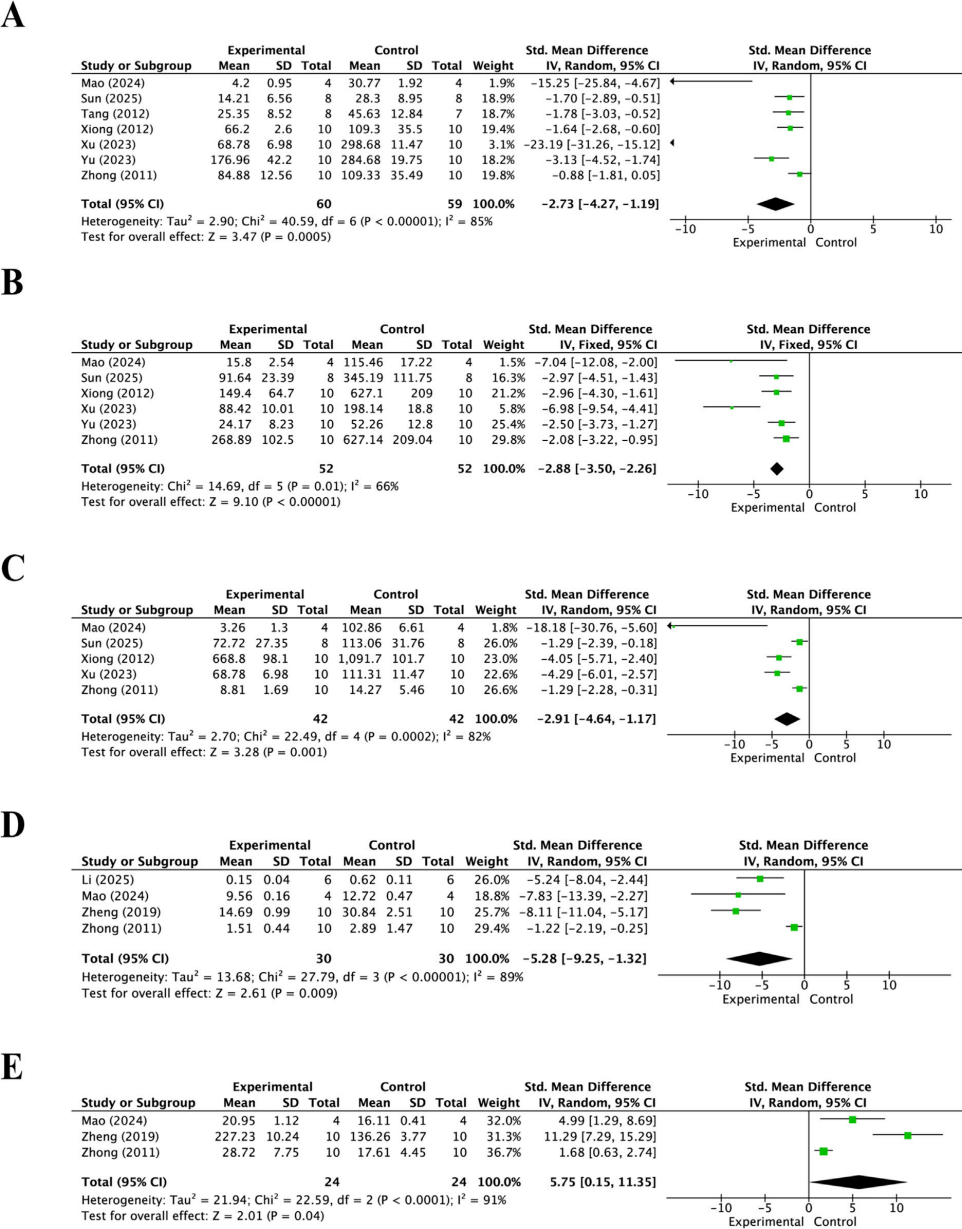
Effects of matrine-type alkaloids on inflammatory cytokines and oxidative stress markers in animal models of ulcerative colitis. **(A)** Forest plot showing the effect of matrine-type alkaloids on TNF-α levels. **(B)** Forest plot showing the effect of matrine-type alkaloids on IL-6 levels. **(C)** Forest plot showing the effect of matrine-type alkaloids on IL-1β levels. **(D)** Forest plot showing the effect of matrine-type alkaloids on MDA levels. **(E)** Forest plot showing the effect of matrine-type alkaloids on SOD activity.

A total of 6 studies were included in the analysis of TNF-α levels. The results showed that, compared with the control group, the matrine-type alkaloid treatment group exhibited significantly reduced TNF-α levels (SMD = −2.88, 95% CI = −3.50 to −2.26, p < 0.001) (Figure 4A). A total of 5 studies were included in the analysis of IL-6 levels. The results showed that, compared with the control group, the matrine-type alkaloid treatment group exhibited significantly reduced IL-6 levels (SMD = −2.91, 95% CI = −4.64 to −1.17, p = 0.001) (Figure 4B). A total of 7 studies were included in the analysis of IL-1β levels. The results showed that, compared with the control group, the matrine-type alkaloid treatment group exhibited significantly reduced IL-1β levels (SMD = −2.73, 95% CI = −4.27 to −1.19, p < 0.001) (Figure 4C).

#### 3.4.4 MDA and SOD

A total of 5 studies were included in the analysis of MDA levels. The results showed that, compared with the control group, the matrine-type alkaloid treatment group exhibited significantly reduced MDA levels (SMD = −3.06, 95% CI = −4.47 to −1.65, p < 0.001) (Figure 4D). A total of 5 studies were included in the analysis of SOD levels. The results showed that, compared with the control group, the matrine-type alkaloid treatment group exhibited significantly increased SOD levels (SMD = 2.63, 95% CI = 1.37 to 3.89, p < 0.001) (Figure 4E).

#### 3.4.5 Effects on body weight

**FIGURE 5:**
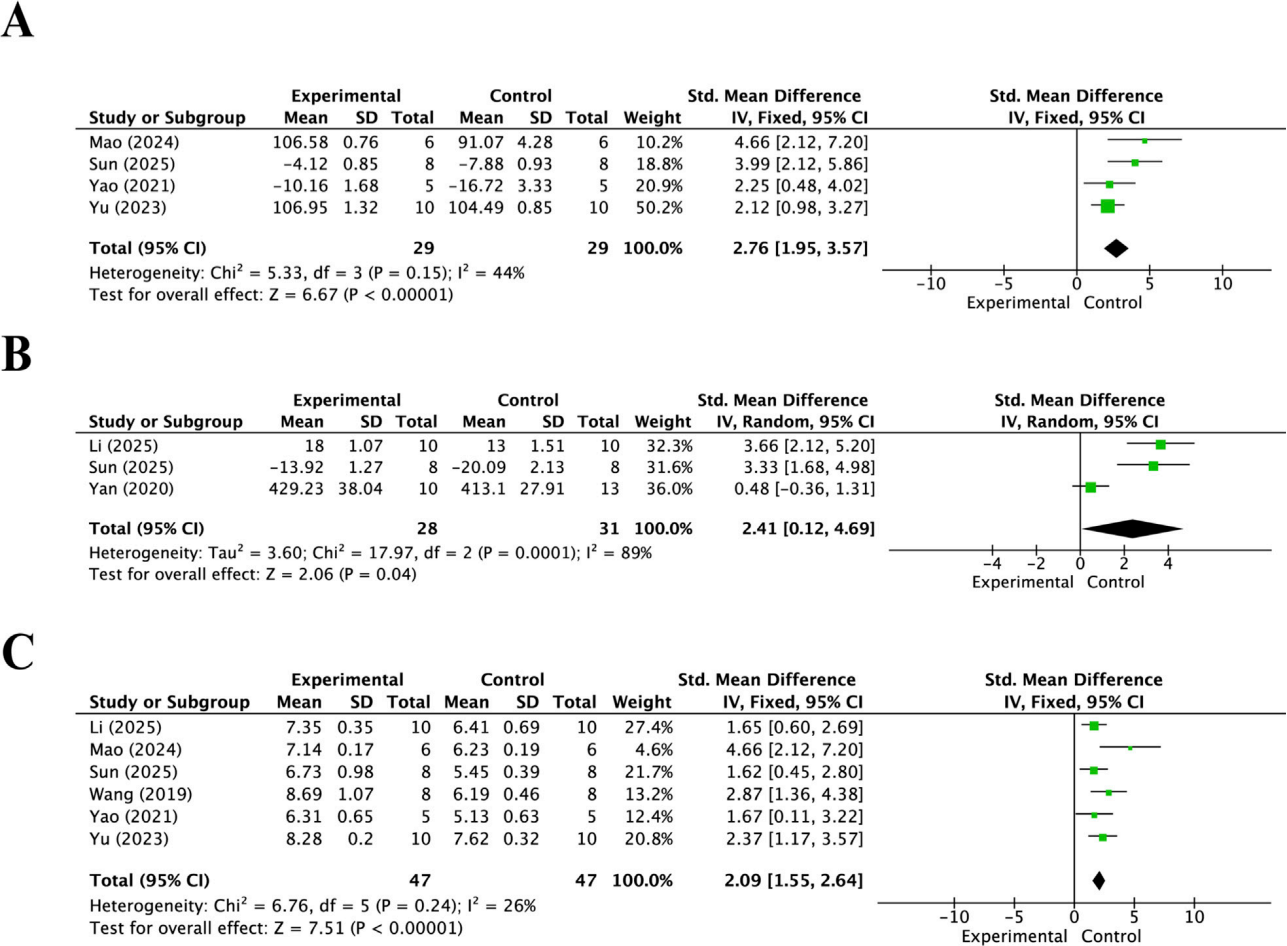
Effects of matrine-type alkaloids on body weight and colon length in animal models of ulcerative colitis. **(A)** Forest plot showing the effect of matrine-type alkaloids on body weight at 7 days. **(B)** Forest plot showing the effect of matrine-type alkaloids on body weight at 14 days. **(C)** Forest plot showing the effect of matrine-type alkaloids on colon length.

A total of 7 studies were included in the analysis of body weight. Among these, 4 studies measured outcomes at 7 days and 3 at 14 days. The results showed that, compared with the control group, the matrine-type alkaloid treatment group exhibited significantly increased body weight at both time points: 7 days (SMD = 2.76, 95% CI = 1.95–3.57, p < 0.001) and 14 days (SMD = 2.41, 95% CI = 0.12–4.69, p = 0.04) (Figures 5A,B).

#### 3.4.6 Effects on colon length

A total of 6 studies were included in the analysis of colon length. The results showed that, compared with the control group, the matrine-type alkaloid treatment group exhibited significantly increased colon length (SMD = 2.09, 95% CI = 1.55–2.64, p < 0.001) (Figure 5C).

### 3.5 Subgroup analysis

Subgroup analyses were performed according to species, model type, sample size, and type of matrine-type alkaloid to explore potential sources of heterogeneity in histopathological scores (Table 2). The results showed that matrine-type alkaloids significantly reduced histopathological scores in both rats (SMD = −2.16, 95% CI: −3.29 to −1.03, I^2^ = 68%, p < 0.01) and mice (SMD = −5.12, 95% CI: −8.34 to −1.90, I^2^ = 88%, p < 0.01). Significant improvements were also observed in both DSS-induced models (SMD = −3.85, 95% CI: −6.04 to −1.67, I^2^ = 81%, p < 0.01) and TNBS-induced models (SMD = −3.24, 95% CI: −5.29 to −1.19, I^2^ = 88%, p < 0.01). When stratified by sample size, significant reductions in histopathological scores were found in studies with sample sizes ≤8 (SMD = −2.87, 95% CI: −4.18 to −1.56, I^2^ = 56%, p < 0.01) and >8 (SMD = −4.31, 95% CI: −7.01 to −1.60, I^2^ = 92%, p < 0.01). In addition, both matrine (SMD = −1.79, 95% CI: −2.52 to −1.07, I^2^ = 38%, p < 0.01) and oxymatrine (SMD = −6.70, 95% CI: −9.62 to −3.79, I^2^ = 73%, p < 0.01) were associated with reduced histopathological scores. Although the pooled effect estimate appeared larger in the oxymatrine subgroup, this finding should be interpreted cautiously because it was based on an indirect subgroup comparison and may be confounded by differences in model type, dosage regimen, route of administration, intervention duration, study quality, and outcome measurement methods. Therefore, the present evidence is insufficient to conclude that oxymatrine is superior to matrine.

**TABLE 2:** Subgroup analysis of histopathological scores in experimental ulcerative colitis models.

Subgroup	SMD (95% CI)	I^2^ (%)	p value
Species			
Rats	−2.16 [−3.29, −1.03]	68	<0.01
Mice	−5.12 [−8.34, −1.90]	88	<0.01
Model			
DSS	−3.85 [−6.04, −1.67]	81	<0.01
TNBS	−3.24 [−5.29, −1.19]	88	<0.01
Sample size			
≤8	−2.87 [−4.18, −1.56]	56	<0.01
>8	−4.31 [−7.01, −1.60]	92	<0.01
Type of matrine-type alkaloid			
Matrine	−1.79 [−2.52, −1.07]	38	<0.01
Oxymatrine	−6.70 [−9.62, −3.79]	73	<0.01

### 3.6 Sensitivity analysis and publication bias

**FIGURE 6:**
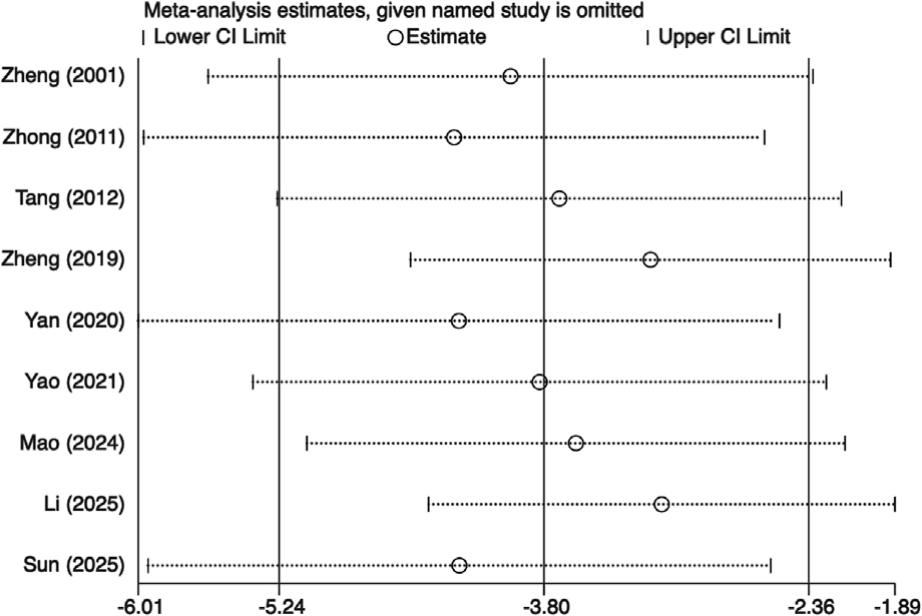
Sensitivity analysis of the effects of matrine-type alkaloids on histopathological scores in experimental ulcerative colitis models using the leave-one-out method.

Sensitivity analysis of histopathological scores showed that sequential omission of each individual study did not materially alter the pooled effect size, and the overall estimate remained stable. The recalculated effect sizes were consistently in the same direction, with the 95% confidence intervals not showing substantial deviation from the overall result, indicating that the pooled finding was not driven by any single study (Figure 6). Publication bias was not formally assessed for most outcomes because fewer than 10 studies were included in each outcome-specific meta-analysis. Although 12 studies reported DAI, the analyses were stratified by time point, and the number of studies within each time-point subgroup was less than 10. Therefore, funnel plots and Egger’s regression test were not performed because such analyses would be underpowered and potentially unreliable. Accordingly, no definitive conclusion could be drawn regarding the presence or absence of publication bias.

## 4 Discussion

This preclinical systematic review and meta-analysis comprehensively evaluated the protective effects of matrine-type alkaloids (matrine and oxymatrine) in animal models of ulcerative colitis. The pooled results suggested that matrine-type alkaloids were associated with improvements in histopathological scores (SMD = −3.47, 95% CI: −4.83 to −2.11), reduced the Disease Activity Index (DAI) at 3 days (SMD = −2.92) and 7 days (SMD = −4.76), increased body weight (7 days: SMD = 2.76; 14 days: SMD = 2.41) and colon length (SMD = 2.09), and markedly decreased pro-inflammatory cytokines, including TNF-α (SMD = −2.88), IL-6 (SMD = −2.91), and IL-1β (SMD = −2.73), as well as the oxidative stress marker MDA (SMD = −3.06), while increasing SOD activity (SMD = 2.63). Overall, these findings suggest that matrine-type alkaloids may exert protective effects in animal models of UC. However, given the substantial heterogeneity across several outcomes, the magnitude and consistency of these effects should be interpreted cautiously. Subgroup and sensitivity analyses provided exploratory support for the direction of the findings but should not be interpreted as definitive confirmation of efficacy across different species, model types, or experimental conditions.

Matrine-type alkaloids were associated with reduced histopathological scores, suggesting potential effects on alleviating structural damage to the colonic mucosa. Histopathological scoring is one of the most critical indicators for assessing tissue injury in UC, and its improvement generally reflects the attenuation of pathological changes such as inflammatory cell infiltration, mucosal edema, crypt destruction, and epithelial loss (Wen et al., 2024). Our findings are consistent with those reported in previous individual animal studies. Li et al. (Li et al., 2019) demonstrated that matrine alleviated TNBS-induced colonic injury in mice by modulating gut microbiota and inflammatory responses. Yao et al. (Yao et al., 2021) reported that matrine improved intestinal barrier integrity and reduced tissue inflammation in DSS-induced colitis. Similarly, Liu et al. (Liu et al., 2022) showed that oxymatrine markedly improved histopathological outcomes in experimental colitis models. Mechanistically, these histological improvements are likely attributable to the combined effects of multiple pathways, including inhibition of inflammatory cell infiltration, attenuation of oxidative stress, restoration of epithelial barrier integrity, and modulation of the immune microenvironment, ultimately leading to reduced tissue damage (Lin et al., 2022).

With respect to disease activity, the pooled results showed that matrine-type alkaloids were associated with reduced Disease Activity Index (DAI) at both 3 and 7 days, suggesting possible beneficial effects during the early and acute phases of inflammation (Mao et al., 2024a). Consistent with this finding, previous studies have reported that oxymatrine (OMT) exerts beneficial effects in animal models of inflammatory bowel disease (IBD), highlighting its potential therapeutic relevance (Zhao et al., 2025). DAI is a composite index that integrates body weight loss, stool consistency, and fecal bleeding, and is therefore widely used to reflect overall disease activity. Notably, no significant difference in DAI was observed at 14 days in the present study, which was not entirely consistent with the results of histopathological scores and inflammatory markers. One possible explanation is that DAI, as a composite endpoint, may be more susceptible to factors such as spontaneous recovery of the model, variations in observation time, and the subjective nature of scoring. Alternatively, matrine-type alkaloids may exert stronger effects during the acute inflammatory phase, whereas their benefits in later stages are more related to tissue repair and molecular regulation, which may not be fully captured by DAI. Taken together, the temporal inconsistency in DAI suggests that different outcome measures may exhibit varying sensitivity across different stages of disease progression, and the disease-activity findings should therefore be interpreted cautiously.

The pooled results showed that matrine-type alkaloids were associated with increased body weight and colon length, suggesting possible improvements in overall disease status. Body weight loss and colon shortening are among the most common phenotypic features in UC animal models and are closely associated with the severity of inflammation, tissue injury, and impaired nutrient absorption (Park et al., 2015; Yan et al., 2009). Previous experimental studies on matrine and oxymatrine have reported similar findings, indicating their ability to improve general condition, alleviate colon atrophy, and promote disease recovery (Liu et al., 2022; Mao et al., 2024a; Zhao et al., 2025). The underlying mechanisms may be related to reduced inflammatory burden, attenuation of mucosal injury, and restoration of intestinal barrier function. Notably, Yao et al. (Yao et al., 2021) demonstrated that matrine improves the expression of tight junction-related proteins and protects intestinal barrier integrity, which may help reduce continuous antigenic stimulation from the intestinal lumen and thereby facilitate the recovery of colon length and body weight.

In terms of inflammatory regulation, the pooled quantitative results showed significant reductions in TNF-α, IL-6, and IL-1β, indicating potential anti-inflammatory activity of matrine-type alkaloids. This finding is highly consistent with the pathophysiology of UC, as these cytokines are key drivers of mucosal inflammation and represent major therapeutic targets in current UC treatments (Dong et al., 2013). The consistent direction of effects across all three cytokines further suggests that the anti-inflammatory action of matrine-type alkaloids is not limited to a single mediator but rather reflects a broader suppression of the inflammatory network. In addition to the pooled cytokine evidence, individual experimental studies and external literature provide narrative evidence for several possible upstream mechanisms. Liu et al. (Liu et al., 2022) reported that oxymatrine alleviates colitis by inhibiting the TLR/NF-κB signaling pathway and suppressing inflammatory dendritic cell activation. In addition, matrine has been shown to exert anti-inflammatory effects through modulation of gut microbiota and PPAR-α-related pathways (Li et al., 2019; Grabacka et al., 2022). Moreover, matrine directly targets the JAK2/STAT3 signaling axis, thereby regulating inflammation, oxidative stress, and bile acid metabolism (Chen et al., 2022). Oxymatrine has also been reported to inhibit the phosphorylation of MAPK pathway components, including ERK1/2, p38 MAPK, and JNK, further contributing to the suppression of inflammatory responses (Mao et al., 2024b). These pathway-related mechanisms provide biological explanations for the observed anti-inflammatory effects, but they are mainly supported by individual studies or external literature rather than directly established by the pooled analysis. Taken together, the pooled evidence supports the anti-inflammatory effects of matrine-type alkaloids as reflected by reduced pro-inflammatory cytokines, whereas upstream mechanisms such as gut microbiota modulation, PPAR-α signaling, JAK2/STAT3, TLR/NF-κB, and MAPK pathways should be regarded as biologically plausible mechanisms requiring further validation.

In addition to inflammatory markers, the pooled quantitative results showed that matrine-type alkaloids were associated with decreased MDA levels and increased SOD activity, suggesting potential antioxidative effects. Oxidative stress is recognized as a key amplifying factor in the pathogenesis of UC, as it not only directly induces epithelial cell damage but also interacts with inflammatory responses to exacerbate mucosal injury (Tian et al., 2017; Rezaie et al., 2007). Notably, the direction of changes in oxidative stress markers in this study was highly consistent with that of inflammatory cytokines, suggesting that the protective effects of matrine-type alkaloids may involve coordinated regulation of inflammatory activation and oxidative injury (Mao et al., 2024a). Previous pharmacological reviews have also highlighted that matrine possesses strong free radical-scavenging activity, regulates redox homeostasis, and suppresses inflammatory signaling pathways (You et al., 2020; Sun et al., 2022). Taken together, the pooled evidence mainly supports anti-inflammatory and antioxidative effects of matrine-type alkaloids in experimental UC, whereas broader pathway-level mechanisms should be interpreted as plausible explanations that require further validation.

Subgroup analyses suggested that matrine-type alkaloids were associated with reduced histopathological scores across species, model types, and sample size strata. In the compound-type subgroup analysis, the pooled effect estimate appeared larger in the oxymatrine subgroup than in the matrine subgroup. However, this finding was based on an indirect subgroup comparison and may be confounded by differences in model type, dosage regimen, route of administration, intervention duration, study quality, and outcome measurement methods. Therefore, the current evidence is insufficient to conclude that oxymatrine is superior to matrine.

Despite these findings, several limitations should be acknowledged. First, all included studies were preclinical animal experiments, and the lack of clinical data limits the direct translational applicability of the results. Second, substantial heterogeneity was observed in several pooled outcomes, which may be attributable to differences in animal species, model induction methods, compound type, dosage regimens, routes of administration, intervention duration, outcome definitions, and methodological quality. Although subgroup and sensitivity analyses were conducted where feasible, the limited number of studies for some outcomes restricted further dose-, route-, duration-, and risk-of-bias-specific analyses, which may have affected the interpretation of the pooled effect estimates. Third, some studies had relatively small sample sizes, and important methodological details were insufficiently reported, particularly regarding random housing, blinding of caregivers and investigators, random outcome assessment, and blinding of outcome assessment. These unclear-risk domains may have introduced potential bias and reduced confidence in the pooled evidence. In addition, variations in the assessment criteria for DAI and histopathological scores may have affected comparability across studies. Furthermore, because most outcome-specific meta-analyses included fewer than 10 studies, formal assessment of publication bias using funnel plots or Egger’s regression test was not feasible. Therefore, potential publication bias or small-study effects could not be excluded. Finally, although a prespecified strategy was used to select a representative dose group in studies with multiple dosage arms, this approach may still have introduced selection bias and influenced the pooled effect estimates. Therefore, dose–response relationships and long-term outcomes could not be fully evaluated and should be investigated in future studies.

## 5 Conclusion

In conclusion, this preclinical systematic review and meta-analysis suggests that matrine-type alkaloids may exert protective effects in animal models of ulcerative colitis. These effects were associated with improvements in histopathological scores and Disease Activity Index (DAI), restoration of body weight and colon length, downregulation of pro-inflammatory cytokines (TNF-α, IL-6, IL-1β) and MDA levels, and upregulation of SOD activity. These findings indicate that the potential protective effects of matrine-type alkaloids may be related to anti-inflammatory, antioxidative, and intestinal barrier-protective mechanisms. However, given the substantial heterogeneity among included studies, unclear risk of bias in several methodological domains, and the absence of clinical evidence, the results should be interpreted with caution. Further high-quality preclinical studies with standardized experimental designs, dose regimens, and outcome measurements are required to validate these findings and facilitate future clinical translation.

## Data Availability

The original contributions presented in the study are included in the article/Supplementary Material, further inquiries can be directed to the corresponding authors.

## References

[B1] Bu S. Cheng X. Chen M. Yu Y. (2025). Ulcerative colitis: advances in pathogenesis, biomarkers, and therapeutic strategies. Pharmgenomics Pers. Med. 18, 219–238. 10.2147/PGPM.S536459 40937422 PMC12420776

[B2] Caba L. Florea A. Cianga P. Drug V. Popescu R. Mihai C. (2025). Genetic and epigenetic factors in ulcerative colitis: a narrative literature review. Genes (Basel) 16 (9), 1085. 10.3390/genes16091085 41010030 PMC12470167

[B3] Chen A. Fang D. Ren Y. Wang Z. (2022). Matrine protects Colon mucosal epithelial cells against inflammation and apoptosis Via the janus kinase 2/Signal transducer and activator of transcription 3 pathway. Bioengineered 13 (3), 6490–6499. 10.1080/21655979.2022.2031676 35220895 PMC8974140

[B4] Cichewicz A. Tencer T. Gupte-Singh K. Egodage S. Burnett H. Kumar J. (2023). A systematic review of the economic and health-related quality of life impact of advanced therapies used to treat moderate-to-severe ulcerative colitis. Adv. Ther. 40 (5), 2116–2146. 10.1007/s12325-023-02488-z 37000363 PMC10130125

[B5] Derong Xu Z. X. Chen L. Xu Li (2023). Effect of oxymatrine on the expression of inflammatory cytokine and intestinal flora in mice with ulcerative colitis. Anhui Med. Pharm. J. 27 (06), 1107–11+277.

[B6] Dong X.-Q. Du Q. Yu W.-H. Zhang Z.-Y. Zhu Q. Che Z.-H. (2013). Anti-inflammatory effects of oxymatrine through inhibition of nuclear factor-kappa B and mitogen-activated protein kinase activation in lipopolysaccharide-induced Bv2 microglia cells. Iran. J. Pharm. Res. 12 (1), 165–174. 24250585 PMC3813192

[B7] Dubinsky M. Bleakman A. P. Panaccione R. Hibi T. Schreiber S. Rubin D. (2023). Bowel urgency in ulcerative colitis: current perspectives and future directions. Am. J. Gastroenterol. 118 (11), 1940–1953. 10.14309/ajg.0000000000002404 37436151 PMC10617668

[B8] Eder P. Łodyga M. Gawron-Kiszka M. Dobrowolska A. Gonciarz M. Hartleb M. (2023). Guidelines for the management of ulcerative colitis. Recommendations of the Polish society of gastroenterology and the Polish national consultant in gastroenterology. Prz. Gastroenterol. 18 (1), 1–42. 10.5114/pg.2023.125882 37007752 PMC10050986

[B9] Feihao Yu T. W. Luan S. Xiong H. Yin Y. Liu D. Wang H. (2023). Mechanistic study of matrine in the treatment of ulcerative colitis Via modulation of memory B cells through the Akt/Mtor signaling pathway. Lishizhen Med. Materia Medica Res. 34 (03), 537–541.

[B10] Fischer B. C. Musengi Y. König J. Sachse B. Hessel-Pras S. Schäfer B. (2024). Matrine and oxymatrine: evaluating the gene mutation potential using *in silico* tools and the bacterial reverse mutation assay (ames test). Mutagenesis 39 (1), 32–42. 10.1093/mutage/gead032 37877816 PMC10851102

[B11] Grabacka M. Płonka P. M. Pierzchalska M. (2022). The Pparα regulation of the gut physiology in regard to interaction with microbiota, intestinal immunity, metabolism, and permeability. Int. J. Mol. Sci. 23 (22), 14156. 10.3390/ijms232214156 36430628 PMC9696208

[B12] Khan H. M. W. Mehmood F. Khan N. (2015). Optimal management of steroid-dependent ulcerative colitis. Clin. Exp. Gastroenterol. 8, 293–302. 10.2147/CEG.S57248 26648749 PMC4648600

[B13] Le Berre C. Honap S. Peyrin-Biroulet L. (2023). Ulcerative colitis. Lancet London, Engl. 402 (10401), 571–584. 10.1016/S0140-6736(23)00966-2 37573077

[B14] Li P. Lei J. Hu G. Chen X. Liu Z. Yang J. (2019). Matrine mediates inflammatory response Via gut microbiota in Tnbs-induced murine colitis. Front. Physiology 10, 28. 10.3389/fphys.2019.00028 30800071 PMC6376167

[B15] Li P. Jin X. Li Y. Shen Y. Wu J. Kang N. (2025). Oxymatrine ameliorates ulcerative colitis Via improving epithelial barrier function through targeting Dsg2. Phytomedicine 148, 157370. 10.1016/j.phymed.2025.157370 41076918

[B16] Lin Y. He F. Wu L. Xu Y. Du Q. (2022). Matrine exerts pharmacological effects through multiple signaling pathways: a comprehensive review. Drug Des. Devel Ther. 16, 533–569. 10.2147/DDDT.S349678 35256842 PMC8898013

[B17] Liu M. Liu F. Pan Y. Xiong Y. Zeng X. Zheng L. (2022). Oxymatrine ameliorated experimental colitis Via mechanisms involving inflammatory Dcs, gut Microbiota and Tlr/Nf-Κb pathway. Int. Immunopharmacol. 115, 109612. 10.1016/j.intimp.2022.109612 36584572

[B18] Mao N. Yu Y. He J. Yang Y. Liu Z. Lu Y. (2024a). Matrine ameliorates Dss-Induced colitis by suppressing inflammation, modulating oxidative stress and remodeling the gut microbiota. Int. J. Mol. Sci. 25 (12), 6613. 10.3390/ijms25126613 38928319 PMC11204106

[B19] Mao N. Yu Y. Lu X. Yang Y. Liu Z. Wang D. (2024b). Preventive effects of matrine on Lps-Induced inflammation in raw 264.7 cells and intestinal damage in mice through the Tlr4/Nf-Κb/Mapk pathway. Int. Immunopharmacol. 143 (Pt 2), 113432. 10.1016/j.intimp.2024.113432 39447411

[B20] Matsuoka K. Igarashi A. Sato N. Mizuno N. Ishii M. Iizuka M. (2025). Clinical characteristics of patients with difficult-to-treat ulcerative colitis: a nested case-control study using a Japanese claims database. Intest. Res. 24 (1), 103–116. 10.5217/ir.2024.00119 40275819 PMC12884122

[B21] Na S.-Y. Choi C. H. Song E. M. Bang K. B. Park S. H. Kim E. S. (2022). Korean clinical practice guidelines on biologics and small molecules for moderate-to-severe ulcerative colitis. Intest. Res. 21 (1), 61–87. 10.5217/ir.2022.00007 35645321 PMC9911265

[B22] Park Y. H. Kim N. Shim Y. K. Choi Y. J. Nam R. H. Choi Y. J. (2015). Adequate dextran sodium sulfate-induced colitis model in mice and effective outcome measurement method. J. Cancer Prev. 20 (4), 260–267. 10.15430/JCP.2015.20.4.260 26734588 PMC4699753

[B23] Qing Tang H. F. Hu H. Shou Z. Liu X. (2012). Effects of oxymatrine injection on expression of Nod2 and Tnf- Α in colonic intestinal mucosa of rats with experimental colitis. Her. Med. 31 (02), 138–141.

[B24] Raine T. Bonovas S. Burisch J. Kucharzik T. Adamina M. Annese V. (2022). Ecco guidelines on therapeutics in ulcerative colitis: medical treatment. J. Crohns Colitis 16 (1), 2–17. 10.1093/ecco-jcc/jjab178 34635919

[B25] Rezaie A. Parker R. D. Abdollahi M. (2007). Oxidative stress and pathogenesis of inflammatory bowel disease: an epiphenomenon or the cause? Dig. Dis. Sci. 52 (9), 2015–2021. 10.1007/s10620-006-9622-2 17404859

[B26] Savelkoul E. H. J. Thomas P. W. A. Derikx LAAP den Broeder N. Römkens T. E. H. Hoentjen F. (2023). Systematic review and meta-analysis: loss of response and need for dose escalation of infliximab and adalimumab in ulcerative colitis. Inflamm. Bowel Dis. 29 (10), 1633–1647. 10.1093/ibd/izac200 36318229 PMC10547237

[B27] Sun Y. Xu L. Cai Q. Wang M. Wang X. Wang S. (2022). Research progress on the pharmacological effects of matrine. Front. Neurosci. 16, 977374. 10.3389/fnins.2022.977374 36110092 PMC9469773

[B28] Sun K. Lin W. Hong Q. Chen S. Li J. Qiu S. (2025). Matrine: a promising treatment for ulcerative colitis by targeting the Hmgb1/Nlrp3/Caspase-1 pathway. Comb. Chem. and High Throughput Screen. 28 (4), 654–663. 10.2174/0113862073292384240209095838 38347801

[B29] Tian T. Wang Z. Zhang J. (2017). Pathomechanisms of oxidative stress in inflammatory bowel disease and potential antioxidant therapies. Oxid. Med. Cell Longev. 2017, 4535194. 10.1155/2017/4535194 28744337 PMC5506473

[B30] Wen C. Chen D. Zhong R. Peng X. (2024). Animal models of inflammatory bowel disease: category and evaluation indexes. Gastroenterol. Rep. (Oxf) 12, goae021. 10.1093/gastro/goae021 38634007 PMC11021814

[B31] Yan Y. Kolachala V. Dalmasso G. Nguyen H. Laroui H. Sitaraman S. V. (2009). Temporal and spatial analysis of clinical and molecular parameters in dextran sodium sulfate induced colitis. PloS One 4 (6), e6073. 10.1371/journal.pone.0006073 19562033 PMC2698136

[B32] Yan X. Lu Q.-G. Zeng L. Li X.-H. Liu Y. Du X.-F. (2020). Synergistic protection of astragalus polysaccharides and matrine against ulcerative colitis and associated lung injury in rats. World J. Gastroenterol. 26 (1), 55–69. 10.3748/wjg.v26.i1.55 31933514 PMC6952295

[B33] Yang Z. Gao S. Yin T. Kulkarni K. H. Teng Y. You M. (2009). Biopharmaceutical and pharmacokinetic characterization of matrine as determined by a sensitive and robust Uplc-Ms/Ms method. J. Pharm. Biomed. Analysis 51 (5), 1120–1127. 10.1016/j.jpba.2009.11.020 20034755 PMC2859310

[B34] Yao H. Shi Y. Yuan J. Sa R. Chen W. Wan X. (2021). Matrine protects against Dss-Induced murine colitis by improving gut barrier integrity, inhibiting the Ppar-Α signaling pathway, and modulating gut microbiota. Int. Immunopharmacol. 100, 108091. 10.1016/j.intimp.2021.108091 34474274

[B35] Yifan Wang H. F. (2019). Effect of oxymatrine mediated by Rhoa/Rock signaling pathway on expression of E-Cadherin and Tgf-Β in ulcerative colitis. Chin. J. Exp. Traditional Med. Formulae 25 (06), 73–80. 10.13422/j.cnki.syfjx.20190638

[B36] Yongai Xiong L. H. Wang M. Su J. (2012). Study on mechanism of oxymatrine in treatment of ulcerative colitis in rats. Chin. J. Exp. Traditional Med. Formulae 18 (08), 152–155. 10.13422/j.cnki.syfjx.2012.08.058

[B37] You L. Yang C. Du Y. Wang W. Sun M. Liu J. (2020). A systematic review of the pharmacology, toxicology and pharmacokinetics of matrine. Front. Pharmacol. 11, 01067. 10.3389/fphar.2020.01067 33041782 PMC7526649

[B38] Zhang L. Gan H. (2021). Secondary Colon cancer in patients with ulcerative colitis: a systematic review and meta-analysis. J. Gastrointest. Oncol. 12 (6), 2882–2890. 10.21037/jgo-21-800 35070415 PMC8748021

[B39] Zhao X. Ye X. Gu Y. Lou Y. Zhou Z. Ji Y. (2025). Oxymatrine for inflammatory bowel disease in preclinical studies: a systematic review and meta-analysis. Front. Med. (Lausanne) 12, 1542953. 10.3389/fmed.2025.1542953 40370726 PMC12075229

[B40] Zhendong Z. Yang L. (2011). Effects of matrine on cytokines and free radicals in the intestinal mucosa of rats with ulcerative colitis. Chin. Med. Biotechnol. 6 (04), 251–254.

[B41] Zheng Z. (2019). Mechanism of oxymatrine alleviating oxidative damage of colonic mucosal cells by regulating autophagy in ulcerative colitis mice. Chin. J. Mod. Appl. Pharm. 36 (16), 2014–2019. 10.13748/j.cnki.issn1007-7693.2019.16.006

[B42] Zheng P. Niu F. Peng Y. Zeng M. (2001). Effect of oxymatrine on dextran sulfate sodium-induced colitis: a preliminary study. J. Gastroenterol. 6 (04), 209–10+22.

