# Supplementary material for: Protective effects and possible mechanisms of matrine-type alkaloids (matrine and oxymatrine) in animal models of ulcerative colitis: a preclinical systematic review and meta-analysis
Source: Front Pharmacol. 2026 Jul 24;17:1870457. doi: 10.3389/fphar.2026.1870457 (PMC13447476; doi:10.3389/fphar.2026.1870457)
Supplement: Supplementary file 1 [file Table1.docx]

# **Supplementary Table S1.** Complete search strategies for all databases

| No. | Database | Complete search syntax |
| --- | --- | --- |
| 1 | PubMed | (("ulcerative colitis"[Title/Abstract] OR "colitis"[Title/Abstract] OR "Colitis, Ulcerative"[Mesh]) AND ("matrine"[Title/Abstract] OR "oxymatrine"[Title/Abstract] OR "Sophora flavescens"[Title/Abstract] OR "matrine-type alkaloids"[Title/Abstract]) AND ("animal"[Title/Abstract] OR "animals"[MeSH Terms] OR "preclinical"[Title/Abstract] OR "preclinical study"[Title/Abstract] OR "mouse"[Title/Abstract] OR "mice"[Title/Abstract] OR "rat"[Title/Abstract] OR "rats"[Title/Abstract])) |
| 2 | EMBASE | ('ulcerative colitis'/exp OR 'ulcerative colitis':ti,ab,kw OR colitis:ti,ab,kw) AND (matrine:ti,ab,kw OR oxymatrine:ti,ab,kw OR 'sophora flavescens':ti,ab,kw OR 'matrine-type alkaloid*':ti,ab,kw) AND ('animal experiment'/exp OR animal*:ti,ab,kw OR preclinical:ti,ab,kw OR mouse:ti,ab,kw OR mice:ti,ab,kw OR rat:ti,ab,kw OR rats:ti,ab,kw) |
| 3 | Web of Science | TS=(("ulcerative colitis" OR colitis) AND (matrine OR oxymatrine OR "Sophora flavescens" OR "matrine-type alkaloid*" OR "matrine type alkaloid*") AND (animal* OR preclinical OR mouse OR mice OR rat OR rats)) |
| 4 | Scopus | TITLE-ABS-KEY(("ulcerative colitis" OR colitis) AND (matrine OR oxymatrine OR "Sophora flavescens" OR "matrine-type alkaloid*" OR "matrine type alkaloid*") AND (animal* OR preclinical OR mouse OR mice OR rat OR rats)) |
| 5 | CNKI | Subject = ("ulcerative colitis" OR "colitis") AND Subject = ("matrine" OR "oxymatrine" OR "Sophora flavescens" OR "matrine-type alkaloids") AND Subject = ("animal" OR "animal experiment" OR "model" OR "mouse" OR "mice" OR "rat" OR "rats") |
| 6 | Wanfang Database | Subject: ("ulcerative colitis" OR "colitis") AND Subject: ("matrine" OR "oxymatrine" OR "Sophora flavescens" OR "matrine-type alkaloids") AND Subject: ("animal" OR "animal experiment" OR "model" OR "mouse" OR "mice" OR "rat" OR "rats") |
| 7 | VIP Database | (Title/Keyword = "ulcerative colitis" OR Title/Keyword = "colitis") AND (Title/Keyword = "matrine" OR Title/Keyword = "oxymatrine" OR Title/Keyword = "Sophora flavescens" OR Title/Keyword = "matrine-type alkaloids") AND (Title/Keyword = "animal" OR Title/Keyword = "animal experiment" OR Title/Keyword = "model" OR Title/Keyword = "mouse" OR Title/Keyword = "mice" OR Title/Keyword = "rat" OR Title/Keyword = "rats") |
| 8 | SinoMed | ("ulcerative colitis"[common field] OR "colitis"[common field]) AND ("matrine"[common field] OR "oxymatrine"[common field] OR "Sophora flavescens"[common field] OR "matrine-type alkaloids"[common field]) AND ("animal"[common field] OR "animal experiment"[common field] OR "model"[common field] OR "mouse"[common field] OR "mice"[common field] OR "rat"[common field] OR "rats"[common field]) |
